# A System of Medicine

**Published:** 1900-03

**Authors:** 


**A System of Medicine.** By Many Writers. Edited by THOMAS CLIFFORD ALLBUTT, M.D., F.R.S. Vol. VII. Pp. xii., 937. Vol. VIII. Pp. xii., 998. London: Macmillan and Co., Limited. 1899.

These two volumes, forming an addition to the number of which it was originally intended that the work should consist, are occupied wholly in respect of the former volume and to about half of the second volume with the discussion of the important subjects of Diseases of the Nervous System, including Mental Diseases. Vol. vii. opens with the completion of the consideration of Diseases of the Spinal Cord. Myelitis is treated at moderate length, and Erb's symptom-complex is dismissed in four lines. Short articles on caisson disease, spastic paraplegia, amyotrophic lateral sclerosis, are given; longer ones on poliomyelitis anterior, acute and chronic, and locomotor ataxy, and a really splendid description of disseminated sclerosis by Dr. Risien Russell, tend largely to emphasize the high tone of the quality of this section of the volume. The late Sir T. Grainger Stewart has contributed articles upon spastic paraplegia, hereditary ataxy and cerebellar ataxy; although neither here nor elsewhere do we find any allusion to Babinski's sign as a token of organic disease of the lateral tracts. Bulbar palsies are treated of by Dr. Beevor with a valuable and instructive fulness.

The section on Diseases of the Brain is introduced by some articles on general cerebral questions, the longest and most important of which is that by Prof. Ferrier on the regional diagnosis of brain lesions: this is particularly valuable from so high an authority, and is well brought up to date. Dr. Charlton Bastian gives his already well-known views on aphasia. Dr. D. B. Lees and Dr. Barlow at last give a full account in an English text-book of simple meningitis, as well as of the tubercular form, in children. Dr. Byrom Bramwell treats of abscess and tumours of the brain, hardly, perhaps, so fully as might have been expected, had it not been for Prof. Ferrier's article before mentioned. Drs. Savage and Goodall discourse separately of general paralysis of the insane in a good but not convincing manner; if this topic was to be handled by two authorities, one should have been an alienist and one a neurologist, considering how long many cases of this disease continue before they need to be certified and placed under the charge of the alienist. The remainder of the volume is devoted to the consideration of several of the functional diseases of the brain, such as epilepsy, only too concisely treated by Sir Wm. Gowers; the difficulty of distinguishing vertigo, narcolepsy and some other conditions from "petit mal" is very great, and deserves a full and close treatment in a standard high-class work like this. The final portion of the volume is contributed by Dr. Risien Russell in several articles on chorea, the "tics," and some other subjects. Chorea, including the various diseases comprehended under that term, is handled in a most satisfactory manner, with all the author's ability and plenitude of learning and research.

Vol. viii. contains the remainder of the articles on Diseases of the Nervous System and the section devoted to Mental Diseases. In the former group there are several valuable articles. Dr. Vivian Poore reiterates his view that craft-palsies are not due to disorder of any hypothetical "co-ordinating centre" in the cortex. Sir William Gowers thinks that the motor cortex of the brain is the seat of the invisible morbid process causing paralysis agitans, a morbid change invisible to us because, perhaps, we have fixed our attention on nerve cells and not upon dendrites. An instructive article on hysteria by Dr. Ormerod follows, and the section is brought to a conclusion by an admirable discourse on neurasthenia by the Editor and Mr. Victor Horsley. Dr. Clifford Allbutt's style finds an appropriate outlet in giving a graphic description of the forms of neurasthenia, which he considers is due neither to "over-excitability" of nervous structures nor to auto-intoxication. It is impossible not to be convinced that this section on Nervous Diseases has done much to raise the position of English Medicine, and to prove how much good and strenuous work is now being done by neurologists in this country. We feel incapable of adequately expressing our thanks to the authors for so splendid a gift to the profession.

The section on Mental Diseases is edited with the help of Dr. Savage, who has joined to himself as co-operators the leading authorities on insanity in this country. Their expositions will appeal rather to the alienist than to the general physician, but among the series of articles composing the section are several which appeal also to the practitioner who is not a specialist in insanity. Of this latter kind are articles on dull and nervous children by Dr. F. Warner, and on night terrors by Dr. Leonard Guthrie. The relations between vice, crime, and insanity are treated of in a most valuable article by Dr. Mercier, whose medico-legal notes in the *Journal of Mental Science* display his authoritative knowledge of this topic. Criminal lunacy in England is treated by Dr. Nicolson, formerly of Broadmoor; and, finally, a very useful article on English Law and Practice of Lunacy is contributed by Dr. Outterson Wood and Dr. Savage. Considering, however, how unfamiliar lunacy law is both to medical men and to solicitors, a fuller exposition of this important subject would have been of greater value.

Nearly five hundred pages of vol. viii. are devoted to Diseases of the Skin, and have been edited with the able help of Dr. Payne, who has written many of the special articles. The names of the writers are all those of men who are known as dermatologists of renown, and they have produced a series of chapters whose merits are beyond all praise.

The article on Erythema is well done, but we think the mention of a new word (erythrodermia) coined by Besnier might have been omitted, as additional names are, to say the least, very perplexing to students, and should be discouraged. It is intended to include extensive areas of reddened skin (usually accompanied by some degree of infiltration) of doubtful nature, such as are met with in the preliminary stages of mycosis fungoides. This condition may be present for many months, but cannot be diagnosed with any certainty as long as other lesions do not present themselves.

Of the different substances which may cause eczema, we cannot see why mustard, turpentine, and croton oil should be called organic irritants, while ammonia and acetic acid come under chemical irritants. We agree that any of these may produce an eruption indistinguishable from eczema; and that it seems impossible to draw a sharp line between artificial and spontaneous eczema. We are of opinion that Unna overstates the general feeling in the profession that the action of bacteria in the skin so commonly causes eczema; and we think with the author of the article that eczema set up by organisms casually alighting on the skin is probably, to say the least, rare; although these organisms would explain two remarkable properties of eczema—namely, its chronic persistence and its local infectiveness.

As the author points out, the term seborrhœa is unmeaning if there is truth in Unna's opinion that in this condition the sebaceous glands do not participate, but that it is the sweat glands which are perverted in their action. The hypothesis of the implication of the sweat glands, however, as stated, receives little support. It seems to us that the scalp, the perineal region, and the foot are well named the bacterial nurseries. They are the parts where saprophytic bacteria are most abundant, where certainly many more than one species vegetate, and from which they may spread to neighbouring parts, giving rise to local inflammations or eczemas. There are many most useful details given as to treatment; but we do not observe any general reference made as to the antiseptic plan almost universally adopted in the present day.

Under the causes of true or inflammatory acne we are pleased to notice a distinction is given between this condition and impetigo, namely, that in acne, all organisms are sometimes absent, or so few in number as to be unimportant—pustles of acne differing in this respect from those of impetigo. Unna denies the activity of the staphylococci entirely, and regards all stages of acne, even the most severe, as due to his acne bacillus. It is also mentioned (which we think is quite correct) that the suppuration of acne follicles is often connected with a seborrhœic process of the skin, or the adjacent scalp, whence microbes are scattered upon the face and shoulders.

The chapter on drug eruptions is very complete, and with these are described the erythemata produced by the injection of antitoxic serum.

A supplement on malarial fever, giving an account of the life-history of the parasite of malaria, and a copious general index, concludes the last volume of Allbutt's system; a work which speaks eloquently for the ability and energy of its distinguished editor, and which will not soon be out of date or forgotten.

